# An Exploration of the Public’s Attitude toward Antibiotic Use and Prescription in Light of the Recent Ministry of Health Regulations: A Cross-Sectional Study in the Kingdom of Saudi Arabia

**DOI:** 10.3390/healthcare10081520

**Published:** 2022-08-12

**Authors:** Haya M. Almalag, Mohammad J. Al-Yamani, Haya F. Alsalloum

**Affiliations:** 1 Department of Clinical Pharmacy, College of Pharmacy, King Saud University, Riyadh 11451, Saudi Arabia; 2 Pharmacy College, Al-Maarefa University, Riyadh 12395, Saudi Arabia; 3 Clinical Pharmacy Unit, Pharmacy Services Department, King Khalid University Hospital, King Saud University Medical City, Riyadh 11461, Saudi Arabia; 4 University Oncology Center, King Saud University Medical City, Pharmaceutical Services, King Saud University Medical City, Riyadh 11461, Saudi Arabia

**Keywords:** knowledge, attitude, antibiotics, Ministry of Health, restrictions

## Abstract

Background: Restricting antibiotic (AB) use with prescriptions and ensuring proper knowledge and attitudes toward AB use is important to reduce antibiotic resistance (ABR). To prevent resistanse problem, several countries have applied prescribing restrictions. Thus, the aim of this work was to re-evaluate the public knowledge and attitudes related to AB use in light of the recent Ministry of Health (MOH) restrictions on AB prescriptions. Methods: A cross-sectional, population-based survey was distributed through various social media platforms. The survey was distributed via email and personal social media. Data were collected and analyzed using appropriate statistical tests. Result: A total of 1333 participants completed the survey. Most participants were female (i.e., 70%), aged 18–24 years old (i.e., 40%), and were aware of the AB restrictions implemented by the MOH. In addition, 77% of participants were aware of the MOH Emergency Call Center (i.e., the 937 Service), but most did not know that it could be used to obtain an AB prescription. Conclusion: Although the knowledge and attitude of the general Saudi population appear better than before, it remains clear that some elements of the population’s knowledge, attitude, and practice need to be strengthened. This could be achieved by utilizing effective channels such as the MOH 937 Service and increased advertising.

## 1. Introduction

Alexander Fleming’s discovery of penicillin in 1928 led to an era of antibiotics (ABs). The discovery allowed humans to fight infections, improve surgery outcomes, undergo chemotherapy, and decrease child mortality [1]. However, antibiotic resistance (ABR) emerged soon after the discovery of ABs. The ability of microorganisms to resist the effects of ABs is called ABR. The continuing development and predictable spread of ABR have seen its base broaden with the arrival of new drugs to the market. Alexander Fleming alluded to AB misuse in his Nobel Prize acceptance speech by saying, “…the time may come when penicillin can be bought by anyone in the shops”. Indeed, three years after this speech, more than one-third of *Staphylococcus aureus* isolates had developed resistance to penicillin.

The major cause of ABR, which is considered a global threat that requires immediate action, is the misuse of ABs. The misuse of ABs is possibly driven by the absence of regulation and a lack of patient knowledge regarding the correct use of ABs. Therefore, implementing policies and constantly evaluating the state of knowledge of populations and attitudes toward using AB is important, as it will allow the identification and management of potential knowledge gaps via effective educational programs. The World Health Organization (WHO) published a Global Action Plan on ABR for all countries to participate in, with the aim to increase overall public awareness about appropriate AB use and ABR. Public awareness of the significant impact of ABR is the first step required to curb its progression.

The literature discusses ABR prevention from five main perspectives, namely AB prescription and monitoring, assessing the knowledge, attitude, and practice (KAP) of AB use, highlighting the misuse or inappropriate use of ABs, AB self-prescription and encouraging appropriate AB use, and increasing ABR awareness. In Saudi Arabia, over-the-counter AB dispensing was an issue prior to 2015 [2]. Thereafter, the Kingdom of Saudi Arabia joined the WHO Global Health Safety Agenda, and in 2017, the WHO published the Saudi National Action Plan developed by the Ministry of Health (MOH) to combat AB-resistant bacteria [3]. As a part of this effort, the MOH launched a campaign in May 2018 to oversee and prohibit the dispensing of ABs without a prescription. In addition, to increase awareness and public acceptance, the MOH Emergency Call Center (i.e., the 937 Service) was launched, allowing patients to have a consultation with a physician and receive an e-prescription through a phone call or via an online system [4].

Population awareness and KAP relating to AB use in Arab countries in general (i.e., Jordan, Kuwait, Lebanon, Iraq, and the United Arab Emirates), and Saudi Arabia in particular, have been described previously [2,5,6,7,8,9,10,11,12,13,14]. However, an assessment is yet to be conducted since the recent regulations and restrictions in Saudi Arabia.

Therefore, in this study, we aimed to re-evaluate and measure the public awareness of AB use in Saudi Arabia and assess the public knowledge of the MOH regulations and the 937 Service in prescribing ABs.

## 2. Materials and Methods

### 2.1. Study Design

**Figure 1:**
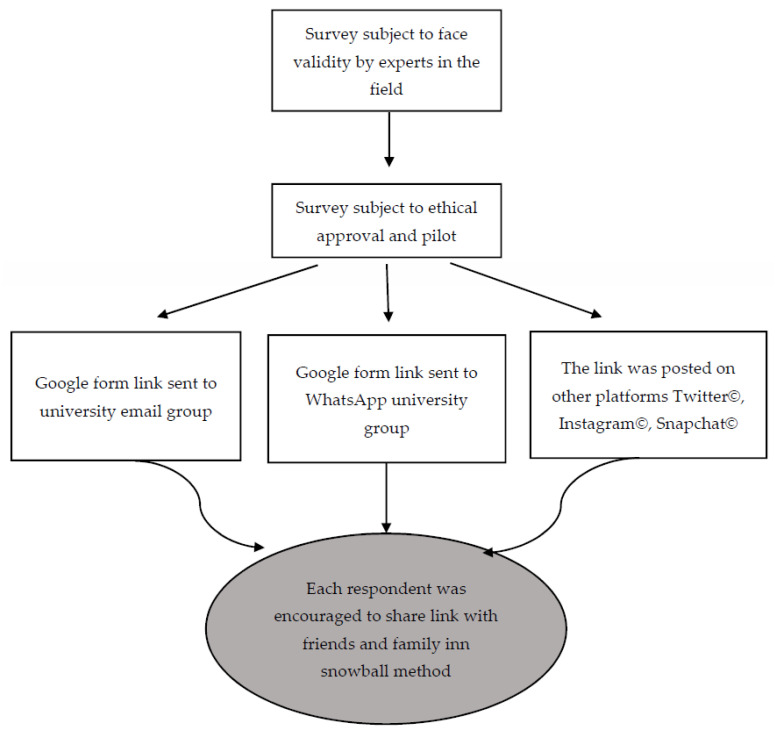
Flow diagram of the methodology.

A cross-sectional survey was designed and conducted among Saudi Arabia’s general population through various social media platforms. This observational study was guided by the Strengthening the Reporting of Observational Studies in Epidemiology (STROBE) statement for cross-sectional studies [15]. A flow diagram of the methodology is shown in Figure 1.

### 2.2. Social Media Platforms

A population-based survey was distributed via university emails and social media platforms (i.e., WhatsApp, Twitter, Instagram, and Snapchat). The private social media accounts of the authors were used to distribute the survey using the snowball method [16].

### 2.3. Participant Eligibility, Exclusion, and Recruitment

All adult participants of Saudi or non-Saudi nationality living in Saudi Arabia and who had agreed to participate voluntarily were recruited between February and April 2021. Participants aged less than 18 years or those living outside Saudi Arabia were excluded. The survey was distributed among the authors’ family, friends, and university email groups via email and WhatsApp, with the participants being asked to forward the survey to as many people as possible. In addition, the Al-Maarefh University administration sent an email to all university students, asking them to participate in the survey. Finally, the research team used their private accounts on Twitter, Instagram, and Snapchat to recruit more participants and request that the survey be forwarded.

### 2.4. Variables, Data Sources, and Measurements

The survey, which was designed and tailored for this study using Google forms, was composed of several sections, including participant demographics, questions assessing participant attitudes toward AB use, participant knowledge of the new MOH restrictions and regulations, and participant awareness of the proper use of ABs as well as the 937 Service and its role in prescribing ABs. The survey underwent a face validity review by two experts in the field of pharmacology at Al-Maarefa University, and it was piloted with ten students that were not among the recruited participants.

### 2.5. Statistical Analyses

Survey response data were collected and analyzed using the Statistical Package for Social Sciences (SPSS) version 27, NY, USA [17]. The means and standard deviations for continuous and normally distributed data were calculated and compared using the independent samples *t*-test. Categorical variables were reported as numbers and percentages and were compared using the chi-square test or Fisher’s exact test, where applicable. Any value that showed a difference was considered significant if it had a *p*-value of <0.05. Factors possibly affecting participant awareness toward AB restrictions categorized as dependent variables were explored by estimating the odds ratio using binary logistic regression. Binary logistic regression was chosen as the dependent variable, and awareness was binary (yes or no). The 95% confidence interval of the odds was also calculated. The reference value in the regression was the lowest group (i.e., lower education, lower age category, strongly disagree responses).

### 2.6. Ethical Approval and Consent

Ethical approval was obtained from the Institutional Review Board of Al-Maarefa University (project number: 06-02032021). Participants were provided with information on the purpose of the study and were asked to provide consent before taking the survey. No participant identifiers were collected during the recruitment process.

## 3. Results

A total of 1333 surveys were fully completed, and the majority of participants were female (i.e., 69.8%) and mostly young (Table 1). More specifically, only 12.5% of participants were above the age of 45 years, while 44.2% were in the age range of 18–24 years (Table 1). Saudi nationals represented 82.7% of the total sample size (Table 1). Only 39% of participants were married, and of those who were married, 90.2% had children (Table 1). The overall participant sample represented an educated group, as less than 1% of participants were illiterate (Table 1). Students represented 36.7% of the total sample size (Table 1), with 57% of those who were not students being employed in either the public or private sector. Unemployed participants represented a small percentage (i.e., 10.9%) of the total sample size (Table 1). There was a high percentage of participants (i.e., 57.7%) who were aware of the AB restrictions imposed by the MOH; furthermore, there was an equal split in the percentage of participants who were and were not aware of the penalties imposed for not complying with the ABs restrictions (Table 1). Surprisingly, only 46.1% of participants were aware of the physician consultations and AB prescription services offered by the MOH 937 Service, despite 76.9% of the participants being aware of the 937 Service being available (Table 1).

When exploring differences in the demographics of participants that were aware or unaware with regards to the MOH AB restriction policy, the results revealed that significantly more male than female participants were aware of the policy (*p*-value < 0.001), with an odds ratio of 1.69 (95% confidence interval: 1.32–2.16) (Table 2). Moreover, as the age of participants grew higher, so did their likelihood of being aware of the MOH AB policy (Table 2). Saudi nationals did not differ from non-Saudi participants in their awareness of the MOH AB restriction policy (Table 2). Married participants were significantly more likely to be aware of the MOH AB policy than non-married participants (*p*-value < 0.001), with an odds ratio of 2.29 (1.81–2.88). Participants with children were significantly more likely to be aware of the MOH AB restriction policy (*p*-value < 0.001), with an odds ratio of 2.84 (2.23–3.63). Compared to participants with a primary or intermediate school education level, university degree holders (*p*-value = 0.025) and high school graduates (*p*-value = 0.006) were significantly less likely to be aware of the MOH AB policy, with odds ratios of 0.45 (0.23–0.91) and 0.37 (0.18–0.76), respectively. When compared to students, participants working in the public (*p*-value < 0.001) and private sectors (*p*-value < 0.001), retired participants (*p*-value < 0.001), housewives (*p*-value = 0.003), and self-employed participants (*p*-value = 0.032) were all significantly more likely to be aware of the MOH AB policy, with odds ratios of 3.72 (2.64–5.23), 2.05 (1.49–2.83), 3.58 (1.94–6.59), 1.81 (1.23–2.70), and 2.28 (1.08–4.83), respectively. Participants that were aware of the 937 Service were significantly more likely to be aware of the MOH AB policy (*p*-value < 0.001), with an odds ratio of 2.75 (2.12–5.58). Regarding the 937 Service and its relation to AB prescriptions, participants that were aware of this relation were significantly more likely to be aware of the MOH AB policy (*p*-value < 0.001), with an odds ratio of 5.41 (4.24–6.90). Finally, participants that were aware of the penalty for violating the MOH AB policy were significantly more likely to be aware of the MOH AB policy itself (*p*-value < 0.001), with an odds ratio of 11.87 (9.10–15.47) (Table 2).

With regards to questions related to KAP (misuse), 42% of participants disagreed or strongly disagreed with the statement (i.e., ‘Whenever I have a cold, I take ABs to make me feel better faster’) (Table 3). However, 61% of participants agreed (43%) or strongly agreed (18%) with the statement (i.e., ‘Whenever I visit the doctor for cold symptoms, I expect him to prescribe me an AB’) (Table 3). Furthermore, 54% of participants (31% agree and 23% strongly agree) claimed to stop taking ABs after feeling better. Regarding AB self-prescriptions, 57% and 82% of participants claim not to advise family members with a cold to take ABs or use leftover ABs for other respiratory infections, respectively. Moreover, more than three quarters (i.e., 75.4%) of participants claimed to check the expiration date of the ABs prior to using them. Moreover, approximately two-thirds (i.e., 66% and 32% agree, and 34% strongly agree) of participants responded that they use ABs according to the enclosed package insert.

**Figure 2:**
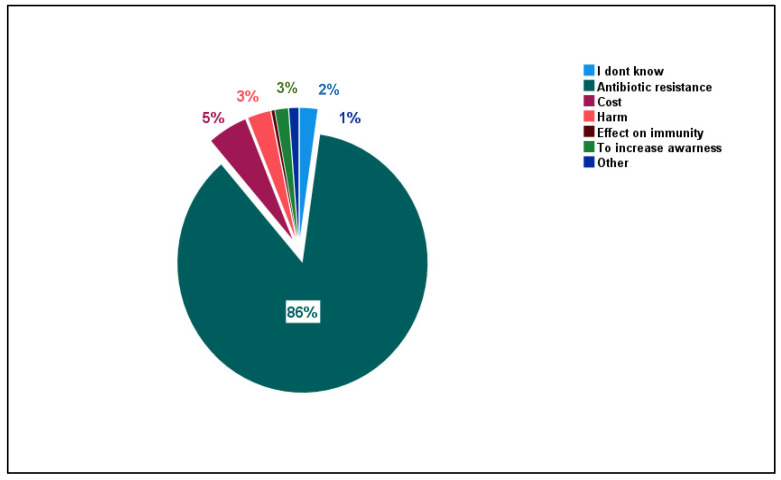
Different reasons given by survey participants to explain the Ministry of Health antibiotic restrictions.

Most participants (i.e., 86.5%) believed that the main reason for banning the sale of ABs without a prescription was the possible development of ABR, while a small percentage of participants (i.e., 5.1%) believed that cost was the main reason (Figure 1). The remaining participants either did not know or had varying perceptions of why the sale of ABs without a prescription was banned, including causing harm, resulting in a negative impact on immunity, resulting in an increase in awareness, or various other reasons (Figure 2).

## 4. Discussion

In this study, we assessed the KAP of patients taking ABs. In addition, we aimed to evaluate the general Saudi population’s knowledge of current MOH restrictions regarding ABs as well as the purpose of these restrictions. With this in mind, we evaluated factors that may affect the population’s awareness so that these factors can be targeted in future population-specific awareness campaigns. We also assessed the population’s knowledge of the very recent MOH Emergency Call Center (i.e., the 937 Service). This assessment may help effectively utilize the tool, ultimately facilitating the accomplishment of the goals set by the WHO and the MOH to prevent and combat ABR.

Participants who were aware of the MOH restrictions were also more aware of the proper use of AB. In addition, these participants tended to disagree more on statements related to AB misuse. These findings could be explained by the fact that participants with higher awareness were older in age. Indeed, although no previous studies reported their results in the same way as done here, there are similarities between our findings and what is reported in the literature. For example, Alqarni et al. [1] reported that knowledge scores were lower in the 18–30-year-old range, a finding that was related to older participants having a higher level of education. Interestingly, however, this was not the case for the results reported here, with education showing no apparent link to KAP.

Given that communities play a significant role in the emergence and spread of ABR, the findings of this study agree somewhat with previous studies regarding AB misuse. A high percentage (i.e., 50.9%) of participants in this survey still expect physicians to prescribe ABs without considering the difference between viral and bacterial infections and how ABs are used in treating infections [6,18,19]. Another finding reported here that confirms previously published work is that participants tend to stop AB therapy whenever they feel better [1]. Furthermore, the degree of the general Saudi population’s awareness was made apparent with some statements associated with AB misuse and self-prescription. Participants generally disagreed with the notion that ABs will make them feel better regardless of the respiratory infection that they might have. Surprisingly, 82% of participants claimed not to use leftover ABs to treat infections; also, 57% of participants claimed not to recommend ABs to ill family members. These findings are in contrast to some previously published studies [1,11,20], and they may be explained by the awareness measures taken by the MOH, or they may be related to other factors that will need to be qualitatively explored. Another interesting finding reported here is that participants know that the MOH restrictions regarding AB use have been put in place because of ABR, a finding that is contrary to that reported by Yagoub et al. [9] in Tabuk, Saudi Arabia, in 2019.

The population studied here generally disagreed with statements of AB misuse, which agrees with some previously published studies. In Saudi Arabia, some studies have revealed that the population had good knowledge and a positive attitude toward AB use. In contrast, a large-scale study investigated the KAP regarding ABs among Hajj pilgrims and reported that the pilgrims had some negative attitudes and poor practices, including acquiring and using ABs without a prescription, sharing ABs, using leftover ABs, and bringing ABs into Saudi Arabia from their country of origin [21,22,23,24]. In addition, several studies have reported AB misuse through retaining leftover ABs for future use [7,25,26]. Finally, it is apparent that participants that were aware of the AB restrictions disagreed more with statements of AB misuse and agreed more with statements of AB proper use.

Assessing the demographic parameters associated with awareness revealed that participants aware of the MOH restrictions were more likely male, of older age (although age can also be related to being married and having children), students, or working in the public sector. Interestingly, we found that being aware of the MOH restrictions was inversely related to the level of education, possibly because students made up the majority of the total sample, and students were the reference group in the analysis. Since the participants were mostly students, our findings are similar to a previous study that reported a knowledge gap being evident among pharmacy students, influencing the quality of AB use in Sri Lanka [27]. Factors affecting awareness could be targeted by awareness campaigns arranged by the MOH or other parties.

The impact of these findings is expected to be high, given that no previous studies have addressed this issue using our methodology. The MOH provides a service whereby residents of Saudi Arabia have 24/7 access to both emergency and standard healthcare via telephone. Given that the participants in this study knew about this service, it could be utilized to impose greater control over AB prescription. However, the general Saudi population needs to be aware that this service can also be utilized for AB prescriptions. Thus, based on our findings, we recommend that the excellent services provided by the MOH, which improve patient healthcare and well-being, be advertised to a greater extent.

This study is not without limitations, as firstly, the sampled population consisted of predominantly young females, thus limiting generalizations. Secondly, the cross-sectional study design may have limited the associations of the explored factors. However, this study involved many participants not limited to a particular region or area, thus representing a diverse population. In addition, the study adds insight into the recent shift in policy related to AB prescription and use.

## 5. Conclusions

The practice of AB prescribing is a great contributing factor to worldwide ABR. Thus, it needs to be frequently evaluated. Although the knowledge and attitudes of the general Saudi population appear much better than before, it remains clear that some elements of the population’s KAP need to be strengthened. This could be achieved by utilizing effective channels such as the MOH 937 Service and increased advertising.

## Figures and Tables

**Table 1:** Demographic parameters of survey participants and a knowledge assessment related to the new Ministry of Health (MOH) antibiotic (AB) restrictions.

N = 1333 Participants		N	%
Gender	**Female**	**931**	**69.8**
	Male	402	30.2
Age	**18–24**	**589**	**44.2**
	25–35	385	28.9
	36–45	192	14.4
	>45	167	12.5
Nationality	Non-Saudi	231	17.3
	**Saudi**	**1102**	**82.7**
Marital status	**Single**	**813**	**61.0**
	Married	520	39.0
Do you have children?	Yes	469	35.2
Education Level	Primary/intermediate school	45	3.4
	High School	346	26.0
	**University and above**	**931**	**69.8**
	Illiterate	11	0.8
Employment	**Student**	**489**	**36.7**
	Public sector employee	247	18.5
	Privet sector employee	234	17.6
	Retired	60	4.5
	Housewife	126	9.5
	Unemployed	145	10.9
	Self-employed	32	2.4
Are you aware of the MOH AB restrictions?	Yes	769	57.7
Are you aware of the penalties for not abiding by these restrictions when getting an antibiotic prescription?	Yes	667	50.0
Are you aware of the MOH 937 Service?	Yes	1025	76.9
Are you aware of the MOH 937 Service and its association with AB prescriptions?	Yes	614	46.1

**Bold** text indicates a higher proportion.

**Table 2:** Demographic parameters of survey participants stratified by their awareness of the MOH AB restrictions and an awareness assessment using bivariate analysis and odds ratios calculated via binary logistic regression.

		Not Aware		Aware		*p*-Value	Odds Ratio	95% CI	*p*-Value
		N	%	N	%				
Gender	Female	429	46.1	502	53.9	<0.001 *	Reference		
	Male	135	33.6	267	66.4		1.690	1.324–2.157	<0.001 *
Age	18–24	321	54.5	268	45.5	<0.001 *	Reference		
	25–35	161	41.8	224	58.2		1.666	1.286–2.160	<0.001 *
	36–45	49	25.5	143	74.5		3.496	2.432–5.024	<0.001 *
	>45	33	19.8	134	80.2		4.864	3.215–7.358	<0.001 *
Nationality	Non-Saudi	102	44.2	129	55.8	0.290	Reference		
	Saudi	462	41.9	640	58.1		1.095	0.823–1.458	0.290
Marital status	Single	406	49.9	407	50.1	<0.001 *	Reference		
	Married	158	30.4	362	69.6		2.286	1.812–2.882	<0.001 *
Do you have children?	No	439	50.8	425	49.2	<0.001 *	Reference		
	Yes	125	26.7	344	73.3		2.843	2.226–3.629	<0.001 *
Education Level	Primary/intermediate school	11	24.4	34	75.6	0.033 *	Reference		
	High School	161	46.5	185	53.5		0.372	0.182–0.758	0.006 *
	University and above	388	41.7	543	58.3		0.453	0.227–0.905	0.025 *
	Illiterate	4	36.4	7	63.6		0.566	0.139–2.305	0.427
Employment	Student	266	54.4	223	45.6	<0.001 *	Reference		
	Public sector	60	24.3	187	75.7		3.718	2.644–5.228	<0.001 *
	Privet sector	86	36.8	148	63.2		2.053	1.491–2.826	<0.001 *
	Retired	15	25.0	45	75.0		3.578	1.943–6.592	<0.001 *
	Housewife	50	39.7	76	60.3		1.813	1.217–2.702	0.003 *
	Unemployed	76	52.4	69	47.6		1.083	0.747–1.570	0.674
	Self-employed	11	34.4	21	65.6		2.277	1.075–4.825	0.032 *
Are you aware of the penalties for not abiding by these restrictions when getting an antibiotic prescription?	No	459	68.9	207	31.1	<0.001 *	Reference		
	Yes	105	15.7	562	84.3		11.868	9.103–15.473	<0.001 *
Are you aware of the MOH 937 Service?	No	189	61.4	119	38.6	<0.001 *	Reference		
	Yes	375	36.6	650	63.4		2.753	2.118–3.578	<0.001 *
Are you aware of the MOH 937 Service and its association with AB prescriptions?	No	431	59.9	288	40.1	<0.001 *	Reference		
	Yes	133	21.7	481	78.3		5.412	4.244–6.902	<0.001 *

* *p* < 0.05 indicates significant differences. CI: Confidence intervals.

**Table 3:** Survey participant knowledge and attitudes toward proper AB use stratified by an assessment of their awareness of the MOH AB policy using bivariate analysis and odds ratios calculated via binary logistic regression.

		Total	Not Aware		Aware		*p*-Value	Odds Ratio	95% CI	*p*-Value
		N (%)	N	%	N	%				
Whenever I have a cold, I take antibiotics to make me feel better faster (misuse)	**Strongly disagree**	**270 (20.3)**	**80**	**29.6**	**190**	**70.4**	<0.001 *	Reference		
	**Disagree**	**294 (22.1)**	**122**	**41.5**	**172**	**58.5**		0.594	0.419–0.842	0.003 *
	Neutral	328 (24.6)	170	51.8	158	48.2		0.391	0.279–0.549	<0.001 *
	Agree	299 (22.4)	133	44.5	166	55.5		0.526	0.372–0.743	<0.001 *
	Strongly agree	142 (10.7)	59	41.5	83	58.5		0.592	0.388–0.905	0.015 *
Whenever I visit the doctor for cold symptoms, I expect him to prescribe me an antibiotic (misuse)	**Strongly disagree**	92 (6.9)	20	21.7	**72**	**78.3**	<0.001 *	References		
	**Disagree**	148 (11.1)	53	35.8	**95**	**64.2**		0.498	0.274–0.906	0.022 *
	Neutral	291 (21.8)	122	41.9	169	58.1		0.385	0.223–0.665	0.001 *
	**Agree**	**569 (42.7)**	**266**	**46.7**	303	53.3		0.316	0.188–0.533	<0.001 *
	**Strongly agree**	**233 (17.5)**	**103**	**44.2**	130	55.8		0.351	0.200–0.613	<0.001 *
I usually stop antibiotic use whenever I feel better (misuse)	Strongly disagree	266 (20.0)	81	30.5	185	69.5	<0.001 *	Reference		
	Disagree	253 (19.0)	91	36.0	162	64.0		0.779	0.540–1.124	0.182
	Neutral	105 (7.9)	47	44.8	58	55.2		0.540	0.339–0.860	0.009 *
	**Agree**	**408 (30.6)**	**196**	**48.0**	**212**	**52.0**		0.474	0.342–0.656	<0.001 *
	**Strongly agree**	**301 (22.6)**	**149**	**49.5**	**152**	**50.5**		0.447	0.316–0.631	<0.001 *
When a family member has a cold, I advise them to take an antibiotic (self-prescription)	**Strongly disagree**	**412 (30.9)**	137	33.3	**275**	**66.7**	<0.001 *	References		
	**Disagree**	**351 (26.3)**	171	48.7	**180**	**51.3**		0.524	0.391–0.703	<0.001 *
	Neutral	260 (19.5)	122	46.9	138	53.1		0.564	0.410–0.775	<0.001 *
	Agree	222 (16.7)	**100**	**45.0**	122	55.0		0.608	0.435–0.849	0.004 *
	Strongly agree	88 (6.6)	**34**	**38.6**	54	61.4		0.791	0.492–1.273	0.334
I use leftover antibiotics for other respiratory illnesses (self-prescription)	**Strongly disagree**	**733 (55.0)**	**297**	**40.5**	**436**	**59.5**	0.182	References		
	**Disagree**	**358 (26.9)**	**165**	**46.1**	**193**	**53.9**		0.797	0.618–1.028	0.081
	Neutral	143 (10.7)	67	46.9	76	53.1		0.773	0.539–1.108	0.160
	Agree	77 (5.8)	27	35.1	50	64.9		1.261	0.772–2.061	0.354
	Strongly agree	22 (1.7)	8	36.4	14	63.6		1.192	0.494–2.877	0.696
I only use antibiotics according to the enclosed package insert instructions (proper use)	Strongly disagree	79 (5.9)	28	35.4	**51**	**64.6**	0.046 *	References		
	Disagree	166 (12.5)	66	39.8	**100**	**60.2**		0.832	0.477–1.450	0.516
	Neutral	204 (15.3)	102	50.0	102	50.0		0.549	0.321–0.939	0.028 *
	**Agree**	**428 (32.1)**	**190**	**44.4**	238	55.6		0.688	0.418–1.133	0.141
	**Strongly agree**	**456 (34.2)**	**178**	**39.0**	278	61.0		0.857	0.521–1.411	0.545
I usually check the antibiotics’ expiry date before I take them (proper use)	Strongly disagree	82 (6.2)	**41**	**50.0**	41	50.0	<0.001 *	References		
	Disagree	95 (7.1)	**50**	**52.6**	45	47.4		0.900	0.498–1.626	0.727
	Neutral	151 (11.3)	89	58.9	62	41.1		0.697	0.406–1.196	0.190
	**Agree**	**328 (24.6)**	152	46.3	**176**	**53.7**		1.158	0.713–1.879	0.553
	**Strongly agree**	**677 (50.8)**	232	34.3	**445**	**65.7**		1.918	1.210–3.042	0.006 *

* *p* < 0.05 indicates significant differences. **Bold** text indicates a higher proportion. CI: Confidence intervals.

## Data Availability

Not applicable.
